# Impact of structured mentorship on professional identity and career development among medical residents: a cross-sectional study in China

**DOI:** 10.3389/fmed.2026.1904776

**Published:** 2026-08-12

**Authors:** Jiayi Tan, Li Jiang, Bo Huang

**Affiliations:** 1 State Key Laboratory of Oral Diseases & National Center for Stomatology & National Clinical Research Center for Oral Diseases, West China Hospital of Stomatology, Sichuan University, Chengdu, Sichuan, China; 2 Emergency General Department, West China Hospital of Stomatology, Sichuan University, Chengdu, Sichuan, China

**Keywords:** career development, medical education and training, mentorship, professional identity, residency standardized training (RST)

## Abstract

**Background:**

Traditional residency training is often characterized by unstructured supervision and frequent clinical rotations, which disrupt the continuity of mentor–mentee relationships and may hinder residents' professional development. This cross-sectional study examined the association of structured mentoring with professional identity and career development among medical residents.

**Methods:**

A cross-sectional survey was conducted among 388 participants, including 172 current residents and 216 graduated physicians, between December 2025 and January 2026. A validated 5-point Likert-scale questionnaire was used to assess structured mentoring, training quality, and career development.

**Result:**

This study investigated 388 early-career physicians, with 84.54% receiving fixed residency mentorship. The mentorship yielded high overall effectiveness (4.39/5) and recognition (4.70/5), excelling in clinical guidance (scores >4.5) but underperforming in research training (3.76/5). Heavy clinical workload (35.06%) and unclear guidance were dominant barriers. The fixed-mentor group showed markedly higher satisfaction (4.39 vs. 3.32, *p* < 0.001), with no significant differences in salary and clinical adaptation. Over 90% supported the program, and 78.87% favored two-way mentor matching as the top optimization strategy.

**Conclusion:**

Fixed longitudinal mentorship was associated with stronger professional identity, better psychological wellbeing, and more favorable early career development among medical residents. Future efforts should focus on strengthening research support, optimizing structured feedback mechanisms, and enhancing mentor development to maximize the effectiveness of mentoring programs.

## 1 Background

Currently, global medical education is transitioning from a knowledge-based model to a competency-based model (1). In contrast to traditional educational models that primarily focus on knowledge acquisition and fixed training duration, Competency-Based Medical Education (CBME) adopts a competency-oriented framework centered on clinical performance. It emphasizes individualized learning pathways based on predefined competency milestones, continuous formative assessment with timely feedback, and outcome-driven training to ensure learners progressively attain the competencies required for safe and independent clinical practice (1, 2). Residency Standardized Training (RST) represents a critical stage in the transition from medical graduate to independent physician. The quality of this training directly influences residents' capacity for lifelong learning and their subsequent professional development (3).

In China, the RST system has been implemented since 2013. As the system has evolved, the focus of medical education reform has shifted from expanding training capacity to improving training quality, making quality enhancement a central priority (4). Traditional RST models primarily rely on unstructured bedside teaching and rotation-based mentorship during clinical rotations, which provide residents with adequate clinical exposure. However, frequent clinical rotations often disrupt the continuity of mentor–mentee relationships, which has been identified as a major contributor to suboptimal educational outcomes in Chinese teaching hospitals (2, 5–8). Recent studies have shown that the lack of continuous mentorship is associated with resident burnout (4, 5), uncertainty in career planning, and poorer performance in residency certification examinations (6). To address these challenges, the Structured Mentorship System (SMS) has been introduced into RST framework. By establishing stable, longitudinal mentor–mentee relationships, the SMS aims to provide comprehensive support for residents' development, including clinical reasoning, research competency, professional integrity, and psychological resilience (7).

Although the importance of mentorship systems has been theoretically recognized in the context of CBME (1, 7, 8), there is still a lack of research to quantify its actual impact on the effectiveness of resident standardized training (RST) and the long-term career trajectories of doctors (2, 9). Researchers mainly focus on the trainees' subjective satisfaction surveys (7). The empirical studies about the correlation between the mentorship allocation and objective outcome indicators are rare (e.g., national graduation examination scores, starting salary of the first job, and career promotion speed) (9).

To investigate the association between structured mentorship and professional identity and career development among medical residents, this cross-sectional study included 388 participants, comprising current residents and practicing physicians. In this study, wellbeing refers to residents perceived psychological wellbeing during training, including emotional support, stress management, resilience, and overall satisfaction with the training experience. Quantitative analyses were conducted to examine the relationships between fixed longitudinal mentorship and clinical reasoning ability, performance in residency certification examinations, and starting salary at first employment. The findings provide empirical evidence for evaluating the effectiveness of ongoing reforms in China's RST system (2) and may also inform the refinement of outcome-oriented medical education models (9). Furthermore, this study offers practical insights for optimizing residency training programs and improving mentorship evaluation and incentive mechanisms (1, 7, 8).

## 2 Materials and methods

### 2.1 Study design and setting

This study used a retrospective cross-sectional design to evaluate the implementation status of mentorship programs and their multi-dimensional impacts on career development among residents in the Chinese Residency Standardized Training (RST) system (9). The questionnaire was independently developed by the research team for this study. Item generation was informed by the study objectives, a review of the relevant literature, and the competency requirements of standardized residency training. An expert panel in medical education and residency training reviewed the questionnaire to establish its content validity and ensure the relevance and clarity of the items before administration. The survey was conducted via a structured online platform from December 1, 2025, to January 31, 2026, covering both active residents in training and physicians who had completed training and entered clinical practice in West China Hospital of Sichuan University, West China School of Stomatology of Sichuan University, West China Second University Hospital of Sichuan University. The content of the survey questionnaire is in Attachment 1.

### 2.2 Participants and sampling

A total of 388 respondents were included in this study. The inclusion criteria were defined as: (1) trainees currently enrolled in a standardized residency program. (2) practicing physicians who had obtained the RST graduation certificate. Among the total sample (*N* = 388), 172 were current trainees (44.33%) and 216 were practicing physicians (55.67%). Demographic analysis showed that the cohort consisted of 132 males (34.02%) and 256 females (65.98%). The participants' educational backgrounds included associate degrees, bachelor's degrees, and graduate degrees.

### 2.3 Data collection instrument

The questionnaire consisted of four core modules:

1. Demographic information: gender, age, highest educational attainment, and current professional status.
2. Mentorship implementation: the key indicator was whether participants had a clear, fixed, full-period mentor (fixed mentorship vs. temporary rotation-based teaching).
3. Training quality evaluation: contributions of mentors in clinical reasoning, research guidance, and career planning were assessed using a 5-point Likert scale (1 = strongly disagree, 5 = strongly agree) (10).
4. Career outcome indicators: annual salary of the first job and the time to promotion to attending physician.

### 2.4 Definition of variables

- Fixed mentorship: A dedicated mentor assigned by the hospital to provide continuous professional guidance and career counseling throughout multiple stages or the entire training period.
- Traditional rotation-based teaching model: *Ad-hoc* preceptors assigned by rotating departments based on scheduling, without a long-term stable supervisory relationship. In this study, the comparison group was the conventional rotation-based training model, in which residents were supervised by different attending physicians during each rotation without assignment to a fixed mentor.
- professional identity outcomes: Objectively measured by systematic clinical reasoning training, doctor–patient communication & humanistic care, hands-on clinical skills training, medical core regulations & medical record quality, career planning & specialty selection, stress relief & emotional support, research initiation & academic writing and ranking in the national qualifying examination and so on (All the content is in the attached questionnaire), which subjectively assessed by Likert scale scores.

### 2.5 Statistical analysis

Statistical analyses were performed by using SPSS version 26.0 (IBM Corp, Armonk, NY, USA). Descriptive statistics were utilized to summarize the data, with quantitative variables expressed as mean ± standard deviation (SD) and qualitative variables as frequencies and percentages. Independent-sample *T*-tests or one-way Analysis of Variance (ANOVA) were used to compare differences in continuous variables, while χ^2^ tests were applied to categorical data. *P* < 0.05 was considered statistically significant.

### 2.6 Ethical considerations

According to the institutional policies of the Ethics Committee of West China Hospital of Stomatology, Sichuan University, this study was exempt from ethical review because it involved an anonymous questionnaire survey, did not collect identifiable personal information, and did not include any invasive procedures or interventions.

## 3 Results

### 3.1 Baseline characteristics of participants

A total of 388 valid responses were included in the analysis. The participants comprised 132 males (34.02%) and 256 females (65.98%), with ages ranging from 22 to 37 years, reflecting an early-career physician population. Regarding the highest educational attainment, 296 (76.29%) held a bachelor's degree, 48 (12.37%) a master's degree, 40 (10.31%) a doctoral degree, and 4 (1.03%) an associate degree, which indicates that most participants had an undergraduate or higher qualification. In terms of professional status, 172 respondents (44.33%) were current residents across various training years (Year 1: 44.19%; Year 2: 27.91%; Year 3: 27.91%), while 216 (55.67%) had completed their Residency Standardized Training (RST) and were in clinical practice.

### 3.2 Fixed mentorship allocation and mentor–trainee interaction

#### 3.2.1 Status of fixed mentorship allocation

Of the 388 participants, 328 (84.54%) were assigned a fixed mentor throughout their residency, including 184 who had completed RST and 144 who were current residents. The remaining 60 participants (15.46%) did not have a fixed mentor and were primarily supervised by rotation-based preceptors, including 32 who had completed RST and 28 who were current residents. The most commonly reported reason for lacking a fixed mentor was the absence of a formal institutional arrangement in the department or training base (40 participants, 66.67%), followed by insufficient mentor resources (8, 13.33%), personal reasons for not applying or matching (8, 13.33%), and other reasons (4, 6.67%).

#### 3.2.2 Frequency and challenges of mentor–trainee interaction

Among the 328 participants with a fixed mentor, the frequency of mentor–mentee interactions was generally high: 185 (56.40%) communicated twice or more weekly, 95 (28.96%) once weekly, and 48 (14.63%) less than once every 2 weeks (Table 1). Among the 328 participants with a fixed mentor, 196 (59.76%) reported no major difficulties in the mentoring relationship. The main challenges were insufficient effective guidance time due to heavy clinical workload (115, 35.06%) and unclear guidance direction attributed to the lack of regular goal setting and feedback (83, 25.30%). Less commonly reported challenges included mismatches in professional interests or personality (31, 9.45%), insufficient mentoring commitment or ineffective mentoring approaches (26, 7.93%), and inadequate mentor competence or outdated knowledge (3, 0.91%) (Table 1).

**Table 1:** Characteristics of mentor–trainee interaction in the fixed mentorship group (*n* = 328).

Indicator	Category	Frequency (*n*)	Percentage (%)
Interaction frequency	≥2 times/week	185	56.40
	Once/week	95	28.96
	Once every 2 weeks	30	9.15
	Once/month	13	3.96
	Rarely	5	1.52
Interaction difficulties (multiple answers allowed)	No major difficulty	196	59.76
	Insufficient guidance time (heavy workload)	115	35.06
	Lack of regular feedback, unclear guidance	83	25.30
	Mismatched interests/personality, poor communication	31	9.45
	Poor mentoring willingness/methods, formal guidance	26	7.93
	Insufficient mentor competence/knowledge update	3	0.91

### 3.3 Evaluation of mentoring effectiveness

#### 3.3.1 Support levels across guidance dimensions

Participants with a fixed mentor reported a mean overall score of 4.39/5 across seven core mentoring domains. The highest score was observed for systematic training in clinical reasoning (4.63/5), followed by demonstration of doctor–patient communication and humanistic care (4.60/5) and hands-on training in clinical skills (4.56/5). These three domains all received mean scores above 4.5. Guidance in research initiation and manuscript writing received the lowest mean score (3.76/5) (Table 2). Residents with fixed longitudinal mentors reported more positive wellbeing experiences, particularly regarding emotional support, confidence in clinical practice, and perceived psychological security during training.

**Table 2:** Evaluation of support levels across mentoring dimensions (*n* = 328, mean score).

Dimension	1 (*n*, %)	2 (*n*, %)	3 (*n*, %)	4 (*n*, %)	5 (*n*, %)	Mean
Systematic clinical reasoning training	3 (1.22)	4 (1.22)	13 (3.66)	69 (20.73)	239 (73.17)	4.63
Doctor–patient communication & humanistic care	2 (1.22)	2 (0.00)	20 (6.10)	77 (23.17)	227(69.51)	4.60
Hands-on clinical skills training	7 (2.44)	1 (0.00)	25 (7.32)	62 (19.51)	233 (70.73)	4.56
Medical core regulations & medical record quality	2 (1.22)	6 (1.22)	25 (7.32)	75 (23.17)	220(67.07)	4.54
Career planning & specialty selection	7 (2.44)	12 (3.66)	33 (9.76)	84 (25.61)	192 (58.54)	4.34
Stress relief & emotional support	6 (2.44)	17 (4.88)	42 (12.20)	59 (18.29)	204 (62.20)	4.33
Research initiation & academic writing	31 (9.76)	28 (8.54)	65 (19.51)	67 (20.73)	137 (41.46)	3.76
Overall average	—	—	—	—	—	4.39

#### 3.3.2 Overall satisfaction and recognition of mentoring role

The mean recognition score for mentoring effectiveness was 4.70/5 (Table 3). Specifically, 79.87% (262/328) reported that mentors were accessible with smooth communication (4.77/5), 76.52% (251/328) confirmed access to complex cases (4.74/5), and 70.12% (230/328) reported structured knowledge building (4.65/5). 72.92% (239/328) reported receiving regular performance feedback (4.65/5). In these four satisfaction surveys, there weren't any scores of 1 or 2.

**Table 3:** Recognition of mentoring effectiveness (*n* = 328, mean score).

Item	1 (*n*, %)	2 (*n*, %)	3 (*n*, %)	4 (*n*, %)	5 (*n*, %)	Mean
Mentor accessibility & smooth communication	0 (0.00)	0 (0.00)	11 (3.35)	55 (16.77)	262 (79.87)	4.77
Access to complex cases	0 (0.00)	0 (0.00)	9 (2.74)	68 (20.73)	251 (76.52)	4.74
Structured professional knowledge building	0 (0.00)	0 (0.00)	16 (4.88)	82 (25.00)	230 (70.12)	4.65
Regular specific feedback on performance	0 (0.00)	0 (0.00)	27 (8.23)	62 (18.90)	239 (72.87)	4.65
Overall average	—	—	—	—	—	4.70

### 3.4 Impacts of mentorship on residency assessment and career development

#### 3.4.1 Career development outcomes

Among physicians who had completed Residency Standardized Training (RST), the mean score for the impact of mentorship on career development was 4.57/5 (Table 4). Overall, 138 participants (75.00%) reported that mentorship provided a strong foundation for employment (mean score: 4.57/5), 129 (70.11%) reported that mentorship enhanced their clinical reasoning and facilitated adaptation to independent clinical practice (4.58/5), and 132 (71.74%) reported that mentor–mentee relationships had sustained positive effects on their professional development (4.55/5).

**Table 4:** Recognition of mentorship impacts on career development (*n* = 184, mean score).

Item	1(*n*, %)	2(*n*, %)	3 (*n*, %)	4 (*n*, %)	5 (*n*, %)	Mean
Mentorship supported successful employment	3 (1.63)	5 (2.71)	14 (7.61)	24 (13.04)	138 (75.00)	4.57
Clinical reasoning accelerated independent practice	2 (1.09)	3 (1.63)	11 (5.98)	39 (21.20)	129(70.11)	4.58
Long-term career benefits from mentor–trainee ties	1 (0.54)	2 (2.09)	23 (13.04)	26 (14.13)	132 (71.74)	4.55
Overall average	—	—	—	—	—	4.57

Annual salary was categorized into three groups: < 150,000, 150,000–250,000, and >250,000. Among physicians with a fixed mentor, 65 (42.76%), 76 (50.00%), and 11 (7.24%) were distributed across the three salary categories, respectively (Table 5). The corresponding proportions among those without a fixed mentor were 15 (46.88%), 15 (46.88%), and 2 (6.25%), respectively. The salary distribution did not differ significantly between the two groups (χ^2^ = 0.191, *P* = 0.909).

**Table 5:** Annual salary distribution for the first job after residency.

Pretax annual salary	Fixed mentorship (*n* = 152)	No fixed mentorship (*n* = 32)	Total (*n* = 184)
< 150,000 CNY	65(42.76%)	15(46.88%)	80 (43.48%)
150,000–250,000 CNY	76(50.00%)	15(46.88%)	91 (49.46%)
>250,000 CNY	11(7.24%)	2 (6.25%)	13 (7.06%)

#### 3.4.2 Comparison of key outcomes between groups

Independent-samples *t* tests and χ^2^ tests were performed to compare outcomes between participants with and without a fixed mentor. The mean training support satisfaction score was significantly higher in the fixed-mentor group than in the no-fixed-mentor group (4.39 vs. 3.32; *t* = 8.62, *P* < 0.001). In contrast, no significant differences were observed in the proportion of participants with a first-job annual salary ≥150,000 CNY (57.24% vs. 53.12%; χ^2^ = 0.191, *P* = 0.909) or in the perceived adaptation to independent clinical practice score (4.59 vs. 4.38; *t* = 0.63, *P* = 0.531) (Table 6).

**Table 6:** Comparison of key indicators between fixed and non-fixed mentorship groups.

Indicator	Fixed mentorship (*n* = 328)	No fixed mentorship (*n* = 60)	Statistic	*P*-value	Interpretation
Training support satisfaction (mean)	4.39	3.32	*t* = 8.62	< 0.001	Fixed mentorship significantly improves satisfaction
First job ≥150,000 CNY (%)	57.24	53.12	χ^2^ = 0.191	0.909	No significant salary difference
Independent practice adaptation (mean)	4.59	4.38	*t* = 0.63	0.531	Similar adaptation, lower satisfaction without mentor

### 3.5 Perceived benefits and optimization suggestions

#### 3.5.1 Ranking of core benefits of mentorship

Among participants with a fixed mentor, accelerated competency development received the highest comprehensive score (4.59/5) (comprehensive score = Σ rank votes × weight / total votes) and was ranked as the most important benefit by 240 participants (73.17%). The remaining benefits were ranked in the following order: risk reduction (3.23/5), clearer career direction (3.14/5), improved access to resources (2.81/5), emotional support (2.46/5), and other benefits (2.07) (Table 7). Residents prioritize mentorship for skill mastery, risk control, and career guidance (11).

**Table 7:** Ranking of core benefits of mentorship (*n* = 328).

Benefit	Comprehensive score	Rank 1 (*n*, %)	Rank 2 (*n*, %)	Rank 3 (*n*, %)	Ranks 4–6 (*n*, %)
Accelerated mastery of core skills	4.59	240(73.17)	51 (15.55)	29 (8.84)	8 (2.44)
Reduced errors and psychological stress	3.23	23 (7.01)	136(41.46)	61 (18.60)	108 (32.93)
Personalized learning & career planning	3.14	42 (12.80)	78 (23.78)	91 (27.74)	117 (35.67)
Better learning and practice resources	2.81	21 (6.50)	43 (13.10)	117(35.67)	147 (44.82)
Emotional support and sense of belonging	2.46	4 (1.22)	19 (5.79)	89 (27.13)	216 (65.85)
Others	2.07	0 (0.00)	4 (1.22)	15 (4.57)	309 (94.21)

#### 3.5.2 Acceptance of mentorship and improvement strategies

Among all 388 participants, 275 (70.88%) considered the mentorship program highly worthy of promotion, 76 (19.59%) supported its promotion with major reforms, 25 (6.44%) considered its value to be limited, and 12 (3.09%) were unsure. No participant expressed opposition to the implementation of the mentorship program.

The most frequently selected measure for improving the mentorship program was two-way matching based on trainee preferences and mentor expertise (306, 78.87%), followed by mentor training (141, 36.34%), protected teaching time linked to performance evaluation (133, 34.29%), standardized mentoring checklists (97, 25.00%), and incentive mechanisms (90, 23.20%) (Table 8).

**Table 8:** Preferences for mentorship optimization strategies (*n* = 388, multiple answers allowed).

Optimization measure	Frequency (*n*)	Percentage (%)	Core demand
Two-way matching by preference and expertise	306	78.87	Improve fit and personalized guidance
Mentor training in teaching and communication	141	36.34	Enhance mentoring quality
Protected teaching time in performance appraisal	133	34.29	Solve insufficient mentoring time
Standardized guidance content checklist	97	25.00	Ensure structured and clear guidance
Incentives and anonymous feedback systems	90	23.20	Strengthen motivation and quality control
Others	4	1.03	—

## 4 Discussion

The findings of this study suggest that fixed longitudinal mentorship is associated with improved training quality, stronger professional identity, and enhanced early career development among medical residents. These findings also provide empirical evidence to inform the optimization of mentorship systems and support the ongoing transition toward a quality-oriented model of residency training.

### 4.1 Core driving role of mentorship in professional identity

The present study demonstrates that mentors received the highest ratings in oversight of medical core regulations & medical record quality (4.54/5) and hands-on clinical skills training (4.56/5). This finding validates the foundational role of fixed mentorship in standardized residency training. Compared with the traditional rotating instructor model relying on *ad-hoc* preceptorship during department rotations, full-period fixed mentorship provides continuous guidance (12). The benefits of continuous mentorship extend beyond the acquisition of technical skills to the systematic development of clinical reasoning. A study in 2025 indicated that mentorship contributes directly to professional identity, professional growth, and patient care quality, although its implementation shows heterogeneous in different institutions (13). The findings of this study suggest that continuous participation in authentic clinical settings under the guidance of a fixed mentor may facilitate the development of clinical reasoning, professional behaviors, and professional competence. Compared with the conventional rotation-based teaching model, fixed mentorship was associated with more favorable educational outcomes in several domains. This finding is consistent with previous studies (13, 14).

### 4.2 Mentorship as a pillar of professional identity and psychological support

A key finding was the high rating for improved professional identity. It helps resident doctors enhance their sense of belonging and reduce the fear of clinical practice, although they face high risks of burnout in the high-pressure environment of medical education (15, 16). Mentors serve not only as academic guides but also as psychological supporters. They cultivate a strong sense of professional mission in residents through role modeling and personalized guidance, which has a huge positive impact on their future careers (17, 18). Contemporary medical research has clarified that mentorship guidance helps in establishing psychological safety for resident physicians and reducing burnout (19, 20). It also indicates that structured mentorship guidance can strengthen the formation of professional identity by integrating values, behaviors, and professional norms into the trainee's self-concept, which is very important for enhancing the self-efficacy and self-worth of resident physicians (11, 21).

Beyond professional identity, the present findings also suggest that longitudinal mentorship may contribute to residents' wellbeing (22, 23). Stable mentor–trainee relationships provide continuous emotional support, timely feedback, and a psychologically safe learning environment, which may alleviate training-related stress and enhance resilience (23, 24). These findings are consistent with previous studies showing that effective mentorship promotes learner wellbeing and supports healthy professional development (22, 23, 25)

### 4.3 Imbalance between research guidance and feedback mechanisms

Despite high overall satisfaction (4.39/5), two key weaknesses were identified. First, research initiation and academic writing scored the lowest (3.76/5). This gap is common in busy clinical settings, where mentors face competing pressures from patient care, teaching, and research. Due to the urgency of clinical duties, research mentoring is often marginalized (26, 27). However, for the more than 60% of residents with master's degrees or higher, research capacity is a core competency for career promotion. Second, the precision of feedback was insufficient. Although residents widely perceived mentors as accessible and specific, evidence-based performance feedback was limited. Effective medical education depends on constructive observation-based feedback (28, 29). Current mentoring remains biased toward general experience transmission rather than precise, tool-based preceptorship. Recent studies indicate that high-quality mentorship requires standardized feedback frameworks and trained mentors, which are currently lacking in many programs (30). This imbalance suggests that mentoring programs often lack standardized evaluation systems and educational frameworks, which limits their effectiveness and scalability.

### 4.4 Associations between mentorship, career development, and early economic outcomes

Although the sample providing salary data was limited, positive trends linking mentorship to early career performance were observed. Among graduated physicians, 57.24% had an annual income more than 150,000 RMB, suggesting a potential association between high-quality mentorship and greater competitiveness in the job market. Recent research indicates that mentorship is associated with improved academic productivity, career advancement, and employment outcomes, particularly in competitive specialties (12, 24). Mentors also provided valuable informal guidance in specialty selection and career planning (31, 32). Despite the mentoring system supporting career decisions and professional paths, this study shows that structured career guidance is still insufficient. Optimized mentor–mentee matching significantly improves career outcomes and satisfaction, and reinforces the importance of personalized mentor strategies (7, 21, 33).

### 4.5 Limitations and future research directions

This study also has several limitations. First, the sample size of 388 was modest, and its cross-sectional design is inherently susceptible to recall bias. Second, data were mainly self-reported, lacking objective comparisons of clinical skills assessment scores between mentored and non-mentored residents. Future studies should adopt multi-center, large-sample randomized controlled designs and explore solutions to current imbalances, such as dual clinical-research mentorship or structured feedback tools (21, 34).

## 5 Conclusion

This study demonstrates that a fixed longitudinal mentorship model has substantial educational value in residency training. It significantly enhances professional identity, professional identity formation, and psychological wellbeing, and it also contributes to early career development. At the same time, in order to enhance the role of a fixed mentorship system, improvements are needed in strengthening research guidance capabilities, implementing structured and feedback-driven teaching models, enhancing mentor training and evaluation systems, and ensuring protected mentoring time. These reforms will support a transition from “formalized mentorship” to “high-quality, outcome-oriented mentorship systems”.

## Data Availability

The raw data supporting the conclusions of this article will be made available by the authors, without undue reservation.
