# Correction: Impact of structured mentorship on professional identity and career development among medical residents: a cross-sectional study in China

**DOI:** 10.3389/fmed.2026.1999534

**Published:** 2026-10-12

**Authors:** Jiayi Tan, Li Jiang, Bo Huang

**Affiliations:** 1 State Key Laboratory of Oral Diseases & National Center for Stomatology & National Clinical Research Center for Oral Diseases, West China Hospital of Stomatology, Sichuan University, Chengdu, Sichuan, China; 2 Emergency General Department, West China Hospital of Stomatology, Sichuan University, Chengdu, Sichuan, China

**Keywords:** career development, medical education and training, mentorship, professional identity, residency standardized training (RST)

In Table 6, Column 2, the percentages reported for “*First job ≥150,000 CNY (%)*” were transcribed incorrectly during table formatting. The correct values, as reported in Section 3.4.2 of the original text, are 57.24% and 53.12%, respectively. The corresponding results are χ^2^ = 0.191 and *P* = 0.909.

The corrected Table 6 appears below:

**Table 6:** Comparison of key indicators between fixed and non-fixed mentorship groups.

Indicator	Fixed mentorship (*n* = 328)	No fixed mentorship (*n* = 60)	Statistic	*P*-value	Interpretation
Training support satisfaction (mean)	4.39	3.32	*t* = 8.62	<0.001	Fixed mentorship significantly improves satisfaction
First job ≥150,000 CNY (%)	57.24	53.12	χ^2^ = 0.191	0.909	No significant salary difference
Independent practice adaptation (mean)	4.59	4.38	*t* = 0.63	0.531	Similar adaptation, lower satisfaction without mentor

The original version of this article has been updated.

